# Successful management of feline pemphigus foliaceus with pentoxifylline and topical hydrocortisone aceponate

**DOI:** 10.1002/vms3.768

**Published:** 2022-02-25

**Authors:** Stefan Hobi, Julia A. Beatty, Jeanine R. Sandy, Vanessa R. Barrs

**Affiliations:** ^1^ Department of Veterinary Clinical Sciences Jockey Club College of Veterinary Medicine and Life Sciences City University Kowloon Hong Kong China

**Keywords:** autoimmune disease, dermatology, feline, pemphigus, skin, treatment

## Abstract

The treatment regimen for feline pemphigus foliaceus (PF), an autoimmune disease caused by auto‐antibodies against proteins of the desmosome junction, usually includes high doses of oral or parenteral immunosuppressive drugs, typically glucocorticoids. This case adds to a growing body of evidence that topical hydrocortisone aceponate is effective for the treatment of feline PF, and demonstrates the practical use of a non‐invasive diagnostic method for histopathology when owners refuse a biopsy to support a clinical diagnosis of PF. Finally, this case highlights an international trend of owner‐initiated treatment of feline infectious peritonitis (FIP) using unlicensed, unregistered drugs.

## Graphical abstract

We describe an alternative treatment approach in a case of feline pemphigus foliaceus with a history of a severe systemic inflammatory disease using a supportive non‐invasive diagnostic technique.

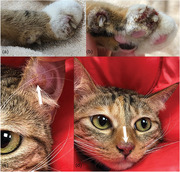

## 1 INTRODUCTION

Pemphigus foliaceus (PF), although rare in most species, is the most commonly reported autoimmune disease in cats (Bizikova & Burrows, 2019a; Scott et al., 2013). Most affected cats are middle‐aged (5–8 years) with no clear breed or gender predisposition and typically present with yellow to brown, adherent crusting skin lesions involving the concave and convex surfaces of the pinnae, dorsal nose, claw folds and circumferentially around the nipples (Bizikova & Burrows, 2019a; Coyner et al., 2018). Pruritus can be variable (Bizikova & Burrows, 2019a; Coyner et al., 2018). Systemic signs are typically mild and non‐specific. These may include fever, lethargy and anorexia (Bizikova & Burrows, 2019b; Jordan et al., 2019; Preziosi, 2019). A definitive diagnosis of feline PF requires histological documentation of epidermal and/or mural follicular pustules containing numerous acantholytic keratinocytes and non‐degenerate neutrophils (Olivry, 2006; Preziosi et al., 2003). In addition, there should be compatible clinical signs. Other causes for crusting and acantholysis, including pyoderma and dermatophytosis, should also be ruled out. Although indirect immunofluorescence to detect IgG anti‐keratinocyte autoantibodies would be ideal to confirm a diagnosis of feline PF (Levy et al., 2020; Olivry, 2006), the diagnostic criteria described above are considered adequate (Bizikova & Burrows, 2019b; Peterson & McKay, 2010; Preziosi, 2019; Preziosi et al., 2003). The prognosis is considered good but most cats with PF require long‐term immunosuppressive treatment (i.e., oral glucocorticoids, ciclosporin, chlorambucil, gold salts, etc.) (Coyner et al., 2018; Peterson & McKay, 2010; Preziosi, 2019). It's important to inform clients about the high relapse risk, either spontaneously or during treatment adjustments. The time until disease control can vary but is on average 3 weeks (Bizikova & Burrows, 2019b; Coyner et al., 2018; Preziosi et al., 2003). In human medicine, drug‐associated PF is subdivided into drug‐triggered and drug‐induced PF (Pile et al., 2021; Wolf et al., 1991). In the latter, there is a good chance of stopping medication without disease relapse after initial treatment and cessation of the offending drug. The former typically needs long‐term treatment similar to spontaneous cases of PF (Wolf et al., 1991). There is evidence that the same classification for PF can be applied to companion animals (Bizikova et al., 2014; Oberkirchner et al., 2011). Various triggers reported in cats include drugs (doxycycline, itraconazole, lime sulphur, others), vaccination, neoplasia (thymoma) and infectious diseases (leishmaniosis) (Affolter & Tscharner, 1992; Imamichi, 2013; McEwan et al., 1987; Rufenacht et al., 2005).

Here we describe a successful therapeutic approach to PF in a cat with severe co‐morbid disease, in which the administration of immunosuppressive doses of systemic glucocorticoids was considered contraindicated. A non‐invasive technique to support our diagnosis was used, which may be useful when full thickness biopsies are not possible.

## 2 CASE DESCRIPTION

A 2‐year old female spayed domestic short‐haired cat with a 2‐month history of non‐resolving skin lesions was presented to the dermatology unit of City University Veterinary Medical Centre, Hong Kong. She had been regularly dewormed, vaccinated, had no travel history and spent the majority of time indoors. Neither the owner nor the other cat in the household had pruritus or skin lesions.

Three months before referral, a presumptive diagnosis of non‐effusive feline infectious peritonitis (FIP) had been made. This was based on consistent clinicopathological findings including fever, weight loss, anorexia and vomiting, mild non‐regenerative anaemia (HCT: 30.2 %, normal range: 30.3–52.3 %; reticulocytes: 19 K/μl, normal range: 3–50 K/μl), hyperglobulinaemia (7.3 g/dl, normal range: 2.8–5.1 g/dl), low range albumin (2.5 g/dl, normal range: 2.3–3.9 g/dl) and decreased albumin/globulin ratio (0.3, normal range: 0.5–1.2). In addition, diagnostic imaging findings included severe enlargement of the mesenteric lymph nodes (3 cm length, median length: 0.8 cm), thickening of the small intestinal muscularis mucosae, and a heterogeneous “mottled” spleen. The presence of pyogranulomatous inflammation with no detectable microorganisms in modified Wright Giemsa stained cytological preparations of fine‐needle aspirate organ biopsies of the small intestine, spleen and enlarged lymph nodes supported a presumptive diagnosis of FIP. There were no skin abnormalities at this time. Independent of veterinary advice, the owner initiated treatment with a 12‐week course of daily subcutaneous injections of an unregistered drug believed to be a nucleoside analogue antiviral (described to be GS441524) and not approved for veterinary use. One week after the presumptive diagnosis of FIP was made, and before commencement of the course of injectable antiviral medication, the cat developed crusting skin lesions on the nose and paws. These skin lesions were empirically treated by another veterinarian with itraconazole (4.8 mg/kg q24 h PO: Itracin^®^; Europharm Lab Co. Ltd., Hong Kong, China), amoxicillin/clavulanic acid (14.5 mg/kg q12 h PO; Amoxyclav^®^; AlfaMedic Ltd., Hong Kong, China), daily topical antiseptic solution containing chlorhexidine gluconate (HiBiSCRUB^®^; Regent Medical Overseas Ltd., Manchester, United Kingdom) and twice weekly topical lime sulphur (Lime Sulfur Dip^®^; Vetoquinol S.A., Lure, France). Ten days later, due to the lack of clinical response the cat was referred to a board‐certified dermatologist for further assessment.

**FIGURE 1:**
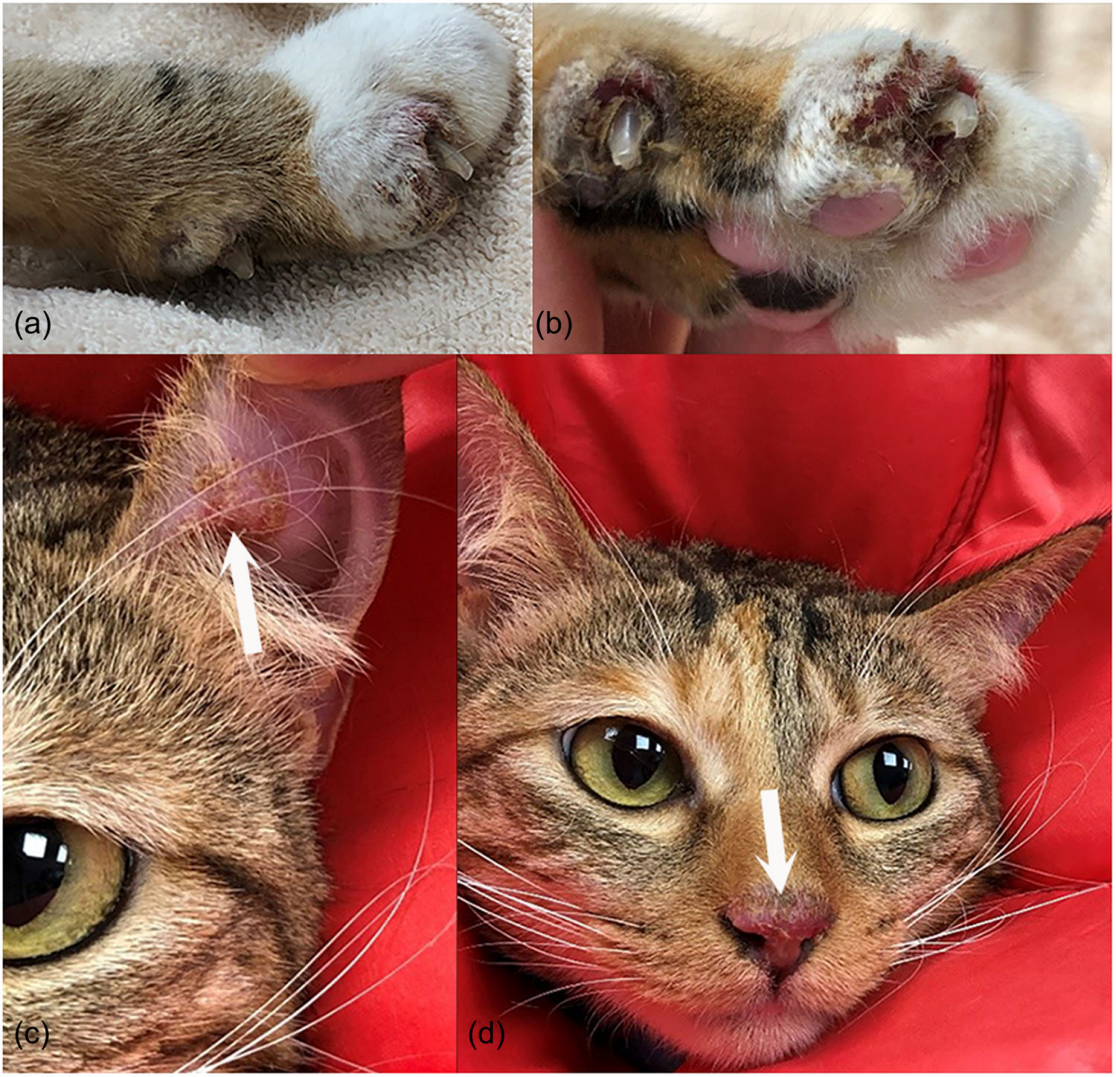
(a) Clinical: initial presentation; hypotrichosis and multifocal crusts with paronychia of P1 and P2. (b) Caseous exudate in the claw folds in addition to paronychia and erosions. Also note the golden crusts involving the paw. (c) Clinical: initial presentation; adherent golden crusts, erosions and erythema on the inner pinnae (arrow). (d) Crusts and erythema on the planum nasale, philtrum and adjacent haired skin (arrow)

**FIGURE 2:**
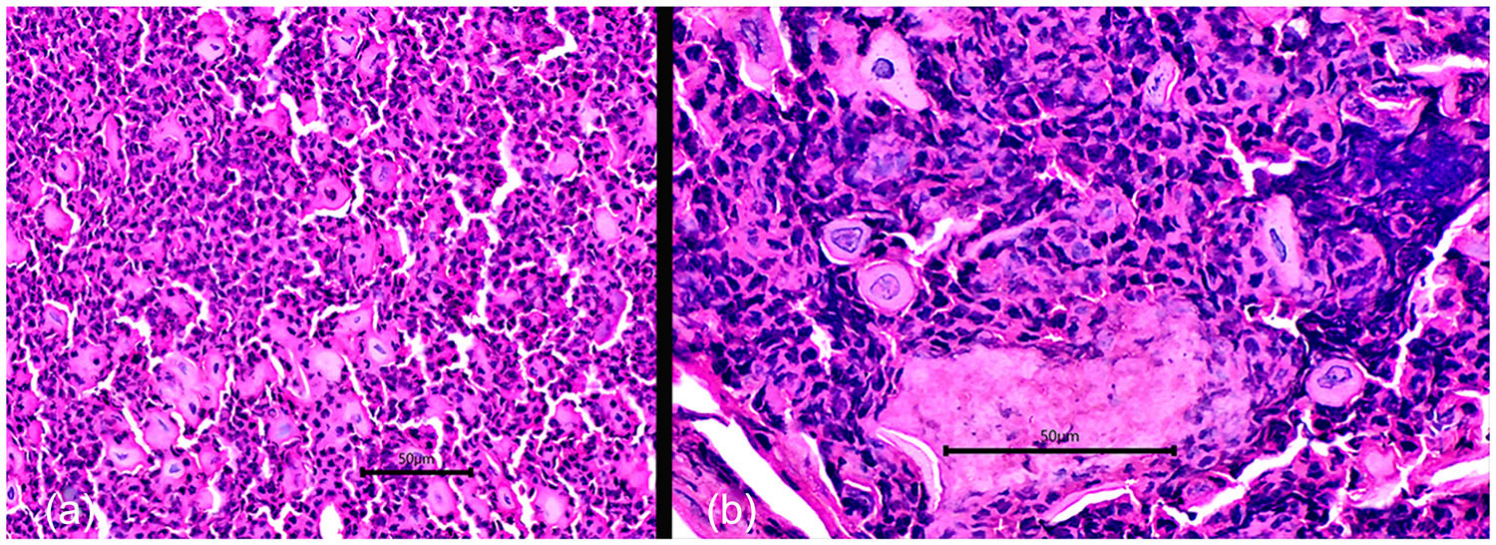
(a) Histopathology (H&E stain × 400 magnification); crust material with abundant neutrophils and scattered angular to rounded acantholytic keratinocytes (arrows). (b) Histopathology (H&E stain × 1000 magnification); crust material; high power to show rounding of acantholytic keratinocytes (arrows) surrounded by a sea of neutrophils

**FIGURE 3:**
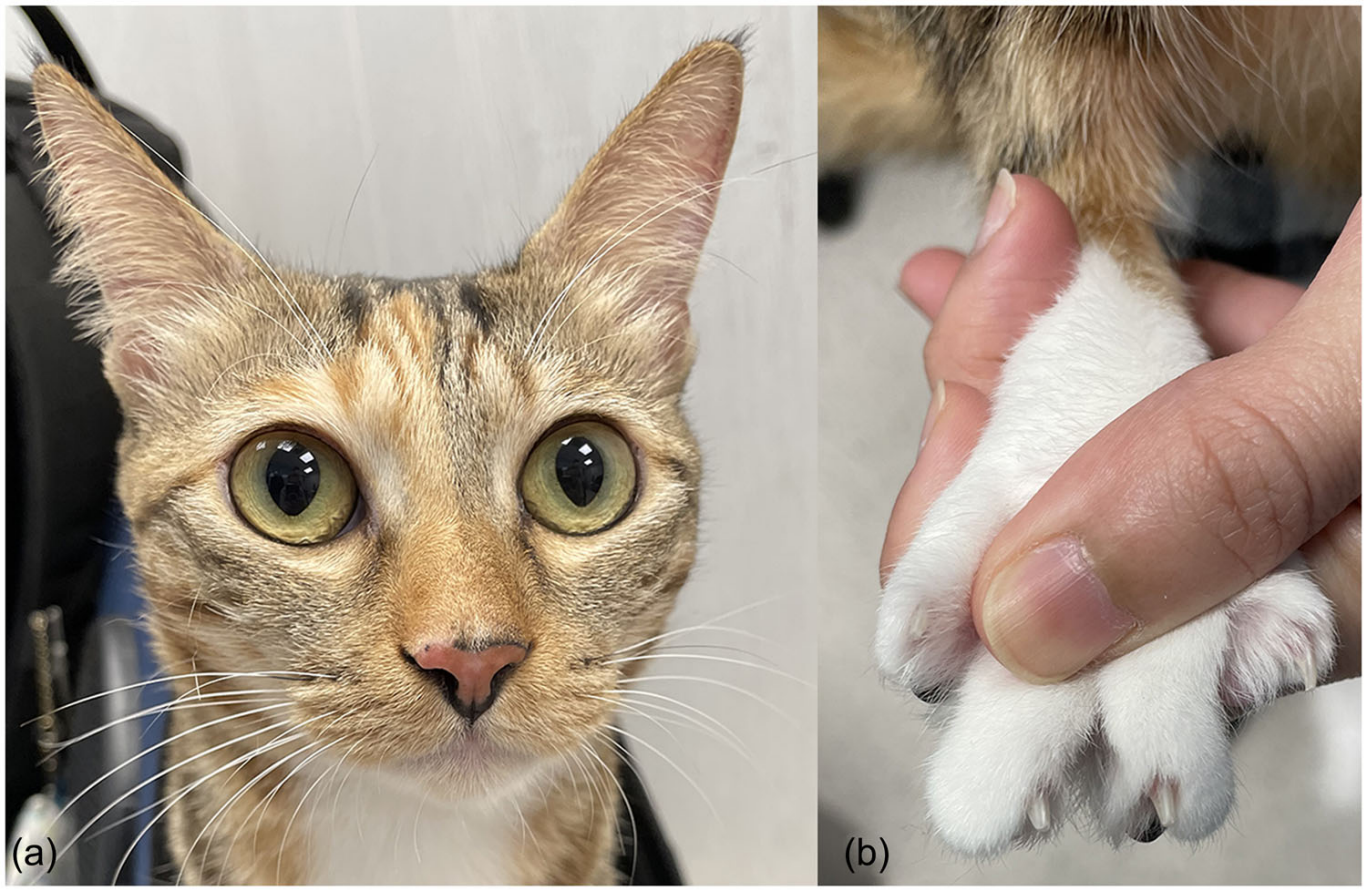
(a), (b) Clinical appearance at the time of writing: current presentation; good general condition, no systemic signs and no pemphigus foliaceus lesions

On physical examination at referral, all vital signs were within normal limits. The cat's body weight was 3.45 kg and the body condition score was 3/9, indicating the cat was underweight. Mild, multifocal erythema and crusting were present on the dorsal nose, concave pinnae and the dorsal, interdigital, haired skin and claw folds of all four paws. Some of the digits were swollen and the cat resented palpation of the digits (Figure 1). Two sharply demarcated, 2 cm large areas of complete focal alopecia with central raised adherent crusts were on the shoulder and mid‐dorsum, in areas of previous antiviral subcutaneous injections. Pruritus was moderate to severe and mainly focused on the affected areas including the aforementioned alopecic locations. Cytological preparations of crusts from the pinnae and affected digits revealed non‐degenerate neutrophils, nuclear streaming and several clusters of acantholytic keratinocytes with an absence of bacterial and fungal organisms. Other dermatological investigations such as skin scraping, trichogram and Wood's lamp examination, from various affected skin areas, were unremarkable. Major differential diagnoses considered were infectious causes (bacterial or fungal organisms, esp. *Trichophyton spp*.), autoimmune causes (PF), cutaneous adverse drug reactions and FIP‐associated dermatitis. Full‐thickness skin biopsies were recommended, but the owner declined because of concerns about sedation and anaesthesia. Instead, multiple crusts from the pinnae and paws were sampled and submitted for histopathology. In addition, dermatophyte culture of hair and crusts as well as an aerobic bacterial culture of surface swabs from the lesions on the paws were performed. A blood test for feline immunodeficiency virus (FIV) and feline leukaemia virus (FeLV) antigen (IDEXX SNAP Combo) was negative. Amoxicillin/clavulanic acid (14.6 mg/kg q12 h PO; Clavulox^®^; Zoetis, Rhodes, Australia) was continued pending the results of the bacterial culture and all topical therapy was discontinued. The antiviral injections were continued by the owner. Three weeks after referral, skin lesions had not improved and new crusting lesions were identified on the dorsum and around the nipples. Cytology from the crusts at the new lesion sites revealed neutrophilic inflammation, clusters of acantholytic keratinocytes and no microorganisms. Cytology of the two focal areas of alopecia was unremarkable. Results from the previous tests included a negative dermatophyte culture and light growth of *Staphylococcus aureus* susceptible to amoxicillin/clavulanic acid. Histopathology revealed multiple large intracorneal crusts composed of abundant intact neutrophils, rounded/angular acantholytic keratinocytes and focal colonies of bacterial cocci at areas of erosions (Figure 2). A Periodic Acid‐Schiff (PAS) stain did not reveal any fungal organisms. A diagnosis of PF was made based on the appearance and distribution of the lesions, supportive histopathology, a negative fungal culture and the lack of response to an antimicrobial to which the cultured *S. aureus* was susceptible. Due to the presumptive FIP diagnosis, immunosuppressive doses of systemic glucocorticoids were avoided and therapy was initiated using topical hydrocortisone aceponate twice daily (one pump per application, applied indirectly via product‐moistened cotton swab) on affected areas (Cortavance^®^; Virbac Limited, Sufflox, United Kingdom), pentoxifylline (26.5 mg/kg q12 h PO; Pentoxifylline 400mg^®^; Oceanside Pharmaceuticals, New Jersey, USA) and omega‐3/6‐fatty acids (767 mg q24 h PO; VetriScience^®^; VetriScience Laboratories; Vermont, USA). At re‐examination 2 weeks later, the skin lesions improved significantly. The cat no longer resented its paws being touched and only a few crusts remained on the front paws and the trunk. The cat was more active and no signs of iatrogenic Cushing's occurred. The owner reported that if the hydrocortisone aceponate application was missed, the crusts immediately relapsed. Cytology of the residual crusts revealed neutrophilic inflammation, no microorganisms and no acantholytic keratinocytes. Pruritus was still present and cetirizine (0.7 mg/kg q12 h PO; Zyrtec^®^; GlaxoSmithKline, Hong Kong, China) was added (Griffin et al., 2012). Two months later, clinical examination revealed a healthy cat, free of systemic signs with complete resolution of crusted skin lesions and pruritus. The 12‐week injection course of the antiviral drug had been completed by the owner during this time. The two well‐demarcated areas of alopecia remained. The topical glucocorticoid was tapered and completely withdrawn after approximately 6 months. The pentoxifylline was subsequently reduced to once daily and withdrawn 4 months later. At the time of writing, 20 months have passed since the initial presentation and 10 months since all medications were discontinued and the cat is clinically normal except for mild gingivostomatitis, with no systemic signs and no relapse of the crusting skin lesions (Figure 3). On repeating serum biochemistry, the only residual abnormality was a mild persistent hyperglobulinaemia (5.6 g/dl, normal range: 2.8–5.1 g/dl). The total serum protein (8.7 g/dl, normal range: 5.7–8.9 g/dl), albumin (3.1 g/dl, normal range: 2.2–4.0 g/dl) and the albumin/globulin ratio had resolved (0.5, normal range: 0.5–1.2). The two alopecic areas persisted.

## 3 DISCUSSION

The diagnosis of non‐effusive FIP can be challenging, since the most common confirmatory testing used by veterinarians in clinical practice is quantitative reverse transcriptase polymerase chain reaction (RT‐PCR) detection of FCoV RNA in effusions, which was not possible in this case (Barker & Tasker, 2020). Quantitative‐PCR of RNA extracts from the organ fine‐needle aspirates of this case, may have facilitated a definitive diagnosis, although a negative test result would not rule out FIP due to the low sensitivity of this technique (Dunbar et al., 2019).

The clinicopathological findings of systemic disease including the fever and diagnostic imaging findings resolved in this case after treatment with the antiviral injections suspected to be the nucleoside analogue GS441524. A recent study demonstrated high efficacy of an unregistered oral multi‐component drug containing GS441524 in curing naturally‐occurring FIP (Krentz et al., 2021). However, the clinical presentation described in the cat in this report could have been caused by other infections (e.g. other viral, toxoplasmosis, atypical bacterial), neoplastic (e.g. lymphoma) or inflammatory diseases, and the compound(s) in the injections administered by the owner could not be confirmed.

The diagnosis of PF in this cat with presumptive concurrent non‐effusive FIP prompted a treatment approach without the use of traditional immunosuppressive drugs that could result in an adverse outcome. We elected to use topical administration of a hydrocortisone aceponate spray, which has been described previously in two cases of feline PF (de Bellis, 2008; Neuber & Shaw, 2011). This drug formulation is licenced for the treatment of inflammatory and pruritic dermatoses as well as atopic dermatitis in dogs. Use in cats is off‐label, although, previous studies support its safety and efficacy (de Bellis, 2008; Neuber & Shaw, 2011; Sauvé, 2019; Schmidt et al., 2012).

In addition, in order to avoid significant suppression of the Th1 immunity by conventional therapy and because of its efficacy in treatment of human pemphigus vulgaris, pentoxifylline was added (Kummari et al., 2020; Marsella & Nicklin, 2000; Takehana et al., 2002). Although not efficacious against FIP, it is known to be well tolerated in cats with FIP (Fischer et al., 2011).

In human medicine, pentoxifylline has been used as an adjuvant medication for the treatment of pemphigus vulgaris since it has been shown to inhibit TNF‐alpha, playing a partial role in the pathogenesis of the disease (Didona et al., 2019; El‐Darouti et al., 2009; Frew et al., 2011; Tham et al., 2020; Zhao & Murrell, 2015). Further studies are needed to evaluate the role of this cytokine in the pathogenesis of feline and canine PF as well as the potential role of pentoxifylline as an effective treatment alternative for this disease.

Both itraconazole and lime sulphur are suspected initiators of PF in cats (Bizikova & Burrows, 2019a, Preziosi et al., 2003). However, as the skin lesions developed before both of these medications were administered and before the first injection of the antiviral drug, it is unlikely these drugs played a role. This was also supported by the calculated Naranjo score (Table 1), based on a questionnaire designed for the determination of a drug to be involved in an adverse event (Naranjo et al., 1981). Provocation, re‐administration of the offending drug, was however not considered due to the possibility of an even more serious reaction. An association with vaccination or a deworming product was considered less likely given the long‐time interval of several months in between the application of these products and the development of PF.

**TABLE 1:** Naranjo score used medications

	Itraconazole				Lime sulphur				Anti‐viral drug			
Questions	Yes	No	?	Sum	Yes	No	?	Sum	Yes	No	?	Sum
Are there previous conclusive reports on this reaction?	1			1	1			1		0		0
Did the adverse event appear after the suspected drug was administered?		–1		–1		–1		–1		–1		–1
Did the adverse reaction improve when the drug was discontinued or a specific antagonist was administered?		0		0		0		0		0		0
Did the adverse event reappear when the drug was re‐administered?			0	0			0	0			0	0
Are there alternative causes (other than the drug) that could on their own have caused the reaction?	–1			–1	–1			–1	–1			–1
Did the reaction reappear when a placebo was given?			0	0			0	0			0	0
Was the drug detected in blood (or other fluids) in concentrations known to be toxic?			0	0			0	0			0	0
Was the reaction more severe when the dose was increased or less severe when the dose was decreased?			0	0			0	0			0	0
Did the patient have a similar reaction to the same or similar drugs in any previous exposure?		0		0		0		0		0		0
Was the adverse event confirmed by any objective evidence?	1			1	1			1	1			1
**Total Score**				**0**				**0**				**–1**

Interpretation all results: doubtful, the reaction was likely related to factors other than a drug.

Viral infections such as COVID‐19, Epstein–Barr virus, human endogenous retroviruses, herpesvirus (human and equine) and canine parvovirus are reported to trigger autoimmune diseases (Ehrenfeld et al., 2020; Favrot et al., 2000; Herder et al., 2012; Nelson et al., 2014; Ruocco et al., 1996; Wang et al., 2005). In addition, FCoV has been directly implicated to cause cutaneous papular to nodular lesions in cats with FIP, characterised histologically by granulomatous inflammation and necrosis. FCoV can be detected in the cytoplasm of macrophages within the dermis in affected skin, using immunohistochemistry (Cannon et al., 2005; Declercq et al., 2008; Redford & Al‐Dissi, 2019). The gross appearance, distribution of skin lesions and histopathological findings in our case were not consistent with FIP‐associated dermatitis. There is increased evidence of a potential association between severe acute respiratory syndrome coronavirus 2 (SARS‐CoV‐2) infection in humans and the rapid development of autoimmune and/or autoimmune dysregulation. Several reaction patterns such as cutaneous rashes, vasculitis, autoimmune cytopenia, anti‐phospholipid syndrome, central and peripheral neuropathy, myositis, myocarditis, Guillain–Barré syndrome, Miller Fisher Syndrome and Kawasaki like disease haven been described (Ehrenfeld et al., 2020; Saad et al., 2021; Talotta & Robertson, 2020). However, since FIP is common and concurrent PF has not previously been reported, such a mechanism is unlikely, yet still possible.

Overall, a spontaneous form of PF is highly unlikely and the final causative trigger is unclear in this case.

The focal temporarily crusted and alopecic lesions which were persistent and non‐responsive to therapy were considered a sequela of the owner‐administered injections since they correlated with the injection sites and this is a well‐known phenomenon in veterinary dermatology (Berrocal, 2004; Gross TL, 2005). Severe irritation and focal areas of necrosis have been reported subsequent to subcutaneous injections of nucleoside analogues such as remdesivir and GS441524 in cats (Izes et al., 2020; Pedersen et al., 2018; Pedersen et al., 2019). In addition, the owner had not administered any topical spot‐on products.

This case also highlights the challenges veterinarians currently face due to a growing widespread practice in Hong Kong and other regions of the use of unregistered, unlicensed drugs that claim to be nucleoside analogues, by owners desperate for a cure of an otherwise fatal disease of their pet (Jones et al., 2021).

Finally, this report also demonstrates that performing histopathology on crusts obtained from representative skin lesions can support a diagnosis of PF when collection of full‐thickness skin biopsies is not possible. Nevertheless, further studies are needed to evaluate the diagnostic utility of this described technique. Other non‐invasive techniques to support a diagnosis include cytological examination of fine‐needle aspirate contents of intact pustules. However, such lesions may be absent when pruritus is present or the pustules rupture due to their thin and fragile nature. Care needs to be taken in interpreting cytological results, since *Staphylococcus spp*. and dermatophytes can cause PF‐like lesions as described in humans and dogs and anecdotally in cats (Olivry & Linder, 2009; Scott, 1980).

In summary, this report describes the first case of feline PF in China and an effective alternative treatment approach consisting of pentoxifylline and topical hydrocortisone aceponate in a patient with a history of a severe systemic infectious disease.

## AUTHOR CONTRIBUTIONS

Stefan Hobi: investigation; writing‐review and editing. Jeanine Sandy: investigation (histopathology); writing‐review and editing. Julia Beatty: investigation; writing‐review and editing. Vanessa Barrs: investigation; writing‐review and editing.

## ETHICS STATEMENT

The authors confirm that the ethical policies of the journal, as noted on the journal's author guidelines page, have been adhered to. No ethical approval was required due to the nature of this case report.

## CONFLICT OF INTEREST

The authors declare no conflicts of interest.

### PEER REVIEW

The peer review history for this article is available at https://publons.com/publon/10.1002/vms3.768.

## Data Availability

The data that supports the findings of this study are available in the supplementary material of this article.
